# Differences in personal care product use by race/ethnicity among women in California: implications for chemical exposures

**DOI:** 10.1038/s41370-021-00404-7

**Published:** 2021-12-24

**Authors:** Hannah N. Collins, Paula I. Johnson, Norma Morga Calderon, Phyllis Y. Clark, April D. Gillis, Amy M. Le, Dung Nguyen, Caroline Nguyen, Lisa Fu, Tiffany O’Dwyer, Kim G. Harley

**Affiliations:** 1 Center for Environmental Research and Children’s Health, School of Public Health, University of California Berkeley, Berkeley, CA USA; 2 California Safe Cosmetics Program, California Department of Public Health, Richmond, CA USA; 3 CHAMACOS, Clinica de Salud del Valle de Salinas, Salinas, CA USA; 4 Healthy Heritage Movement, Riverside, CA USA; 5 California Healthy Nail Salon Collaborative, Oakland, CA USA

**Keywords:** Personal care products, Cosmetics, Health disparities, Chemical exposure

## Abstract

**Background:**

Personal care products may contain many chemicals, some of which are suspected endocrine disrupters. This is an important source of chemical exposure for women, but little is known about how chemical exposure differs among different races/ethnicities.

**Objective:**

This study examines differences in personal care product use among Black, Latina, Vietnamese, Mixed Race, and White women in California.

**Methods:**

We used a community-based participatory process to create and administer a personal care product usage survey to 321 Black, Latina, Vietnamese, Mixed Race, and White women. We used multivariate regression models with pairwise comparisons to examine the frequency of product use by race/ethnicity.

**Results:**

We found distinct trends of personal care product use by race/ethnicity: Latina women typically used makeup most frequently; Black women used certain hair products or styles most frequently; and Vietnamese women were most likely to use facial cleansing products compared to other races/ethnicities. Latina and Vietnamese women were less likely to try to avoid certain ingredients in their products.

**Significance:**

These findings can help estimate disparities in chemical exposure from personal care product use and complement future research on health inequities due to chemical exposures in the larger environmental and social context.

## Introduction

Use of consumer products, including personal care products, is an important potential source of chemical exposure to women and girls. Use of soaps and shampoos, makeup, fragrances, and hair and skincare products is virtually ubiquitous. One small study found that the average woman uses 12 different personal care products containing 168 unique chemicals each day [1]. Although the U.S. Food and Drug Administration (FDA) is the regulating body for personal care products sold in the U.S., it has limited legal authority to require safety testing and does not subject personal care products to the approval processes used for foods and drugs [2]. While the European Union places restrictions on 1328 chemicals from use in cosmetics due to concerns about cancer, genetic mutation, or reproductive or developmental harm [3], the FDA restricts only 11 chemicals in cosmetics [4].

Chemicals of concern in personal care products include suspected endocrine disruptors, such as phthalates (an ingredient in fragrances, nail polish, and makeup), parabens (preservatives in various types of personal care products), triclosan (an antimicrobial agent) and benzophenone-3 ((BP-3), a sunscreen agent), and carcinogens, such as 1,4-dioxane and formaldehyde [5–13]. Human exposure to these chemicals can occur through dermal absorption of products applied topically or through inhalation or ingestion of products during application or wear [14]. Several ingredients or unintentional contaminants found in personal care products have been linked with negative health impacts for women such as cancer [13], breast cancer susceptibility [15–23], and reproductive harm including shortened menstrual cycles, lower antral-follicle counts [24], and altered timing of puberty [25]. Although we know that women generally use a great number of personal care products, less is known about how exposure differs by race/ethnicity. A study in California examined personal care product use among 374 African American, Asian, Latina, and White mothers and found that African American women used permanent chemical hair products such as straighteners or relaxers more frequently than the other groups, though they used shampoo, conditioner, and hair dye less frequently [26]. Asian women used more skincare products and fewer makeup items and deodorants, while White women used more sunscreen [26].

European and White beauty standards can impact personal care product use for women of color as they may use products to achieve straighter hair, lighter skin, or be targeted in marketing campaigns for other products that promote “mainstream beauty norms.”[12] Women of color also experience the co-occurrence of other social and environmental risk factors [12]. There is a growing concern that Black women, in particular, may be disproportionately exposed to potentially harmful chemicals in personal care products. A recent report suggested that African American women spend more than other Americans on personal care products but have fewer choices for safer products and a previous study found that there were significant differences in personal care product use between non-Hispanic Black and White women, particularly in hair product use [27, 28]. Data from the nationally-representative National Health and Nutrition Examination Survey (NHANES) show that African Americans have the highest urinary concentrations of phthalates and parabens, followed by Mexican Americans [29]. Non-Hispanic Whites have the lowest levels of these chemicals [29]. NHANES data also show that Asians have the highest concentrations of triclosan [30]. Additionally, a survey of 301 African American, African Caribbean, Hispanic, and White women in New York City found that African Americans and African Caribbeans used the largest number of hair products and that 69% of the products used contained endocrine disrupting chemicals (EDCs), including parabens [31]. A study on the hormonal activity of commonly used Black hair products found that all of the examined products had estrogen agonist properties, a concern for health outcomes associated with estrogen disruption [32]. Previous studies have also looked at the prevalence of chemical hair straightening, with one finding that in a cohort of African American women adolescence may be a time of increased usage and potential exposure among African American women [33]. Another analysis of NHANES data found that Black women were more likely than White or Latina women to use scented feminine hygiene products, such as douches or feminine sprays, and that use of these products was associated with higher urinary metabolites of diethyl phthalate [34].

There is less research on personal care product use among Asian and Latina women. Some Asian women, particularly Vietnamese women, have higher than average exposures to nail salon products due to their occupation [35], but there is little information about their own personal use of cosmetics and other products. One published study showed that a higher level of acculturation of Chinese women in Boston was associated with higher use of personal care products, but they only examined nine products [36]. Use of skin lightening creams is prevalent in both Asian and Latina women [37, 38], and there have been documented cases of people exposed to mercury through skin lightening cream imported from Mexico [38]. Thus, it is possible that Asian and Latina immigrant women may be buying or importing beauty products from their home countries that contain other chemicals not usually found in mainstream American products, but there is little research to examine this concern. Only one recent study examined detailed personal care product usage in a diverse sample of women in California, documenting several differences in product usage among Black, White, Latinx, Asian, and Multi-Racial women [39].

There are few studies to date that look at detailed personal care product use, particularly in communities of color. Given the exposure and potential health implications of increased product use, more information is needed about personal care product use among specific communities of color, to better estimate risk from exposure and target interventions to minimize risk. Our objective was to characterize trends of personal care product use in order to better understand disparities of chemical exposure among Black, Latina, Vietnamese, Mixed Race and non-Hispanic White women in California. To do this, we surveyed more than 300 women in California about their personal care product use.

## Materials and methods

### Community-based participatory process

Survey planning, design, and implementation was a collaborative process among the three community-based partners and two scientific partners that form the Chemical and Personal Care: Asian, Black and Latina Exposure (CAPABLE) Study. The first community group, Healthy Heritage Movement (HHM), works primarily with Black women in the Inland Empire (San Bernardino County and Riverside County) and Los Angeles County of Southern California. The second, California Healthy Nail Salon Collaborative (CHNSC), works with Vietnamese nail salon workers throughout California, but focused on Los Angeles and Orange Counties for this study. The third group, Clinica de Salud del Valle de Salinas (CSVS), works with Latinos in the Salinas Valley, California. The scientific partners were the California Department of Public Health’s (CDPH) Safe Cosmetics Program and University of California, Berkeley’s Center for Environmental Research and Children’s Health (CERCH). Survey design was informed by focus groups in each community, which involved 7–9 community members and was led by a representative from the community organizations who identified as the same race/ethnicity as the participants. Cognitive interviews (two per community) were conducted to test the understanding and feasibility of the survey prior to administration.

### Study population

Eligible participants were at least 18 years old, living in California, and spoke either English, Spanish, or Vietnamese. Survey recruitment and administration was conducted in person by staff from the community partner organizations. Each community partner recruited a convenience sample of 75–100 participants. Recruitment locations were decided by each community partner, depending on what was most appropriate in their community. HHM recruited primarily Black women from churches, health fairs, a hair show, and through collaboration with community partner organizations in San Bernardino County, Riverside County, and Los Angeles County, California. CHNSC recruited Vietnamese women from nail salons, churches, temples, and colleges in Los Angeles County and Orange County, California. CSVS recruited Latina women from health clinics, schools, and Special Supplemental Nutrition Program for Women, Infants, and Children offices in Salinas, California. White women were recruited by all community partners at these same locations, as well as in Contra Costa County at schools and medical offices. Informed consent was obtained from all participants, who received a $20 incentive coupon upon completion. Surveys were conducted on paper and self-administered. Human subjects approval was received from California Health and Human Services Agency’s Committee for the Protection of Human Subjects.

Surveys on usual personal care product use were completed by 321 Black, Latina, Vietnamese, and non-Hispanic White women between April and December 2019. We limited the study population to Black, Latina, Asian, and White women, including women who identified as more than one of these race/ethnicities (Mixed Race). Women who checked more than one race/ethnicity were categorized as Mixed Race. We excluded two women who were missing race/ethnicity information and one woman who identified as American Indian/Alaska Native for a final sample size of 318.

### Measures

The 40-question survey asked about frequency of personal care product use, what brands participants favor, where they shop, whether they avoid certain ingredients, if they bring or buy products bought from other countries, and demographic information. The survey asked about 81 specific product types that were grouped into six categories of personal care products: makeup, hair, feminine hygiene, skincare, nail, and deodorant/fragrance. Each question asked about the frequency of use of a particular personal care product: every day, 5–6 days per week, 2–4 days per week, 1 day per week, 2–3 times per month, 1 time per month, a few times per year, or never. In addition to product use, the survey asked “Do you ever use any personal care products that you or someone else brings from another country?” and “Do you ever buy personal care products in the U.S. with labels not in English?” with an open-ended follow up question of which country they come from. Lastly, the survey asked: “Are there ingredients that you try to avoid in your personal care products?” and “How do you choose which personal care products to buy?”. Surveys were translated by CSVS and CHNSC staff into Spanish and Vietnamese, respectively.

Women were asked to self-identify their race/ethnicity (White, African American/Black, Latina/Hispanic, Vietnamese, Other Asian, American Indian/Alaska Native, or Other) and could check all that applied. Information on education, income, and other basic demographics was also collected. Most women answered all questions, but the sample size ranged from 305 to 318 for questions on personal care product use.

### Statistical analysis

All survey data were analyzed using STATA version 15.1 [40]. We compared basic demographic characteristics among the different racial/ethnic groups using Chi-square tests. For ease of interpretation, product frequency of use was converted from a categorical variable to a continuous variable of number of times per week to facilitate the comparison of means across groups. We converted “every day”, “5–6 days per week”, “2–4 days per week”, “1 day per week”, “2–3 times per month”, “1 time per month”, “a few times per year”, and “never” to 7, 5.5, 3, 1, 0.5, 0.25, 0.1, and 0 times per week, respectively. For frequently used products (>0.5 times per week on average), we compared mean use per week across racial/ethnic groups as a continuous variable. To control for sociodemographic differences between communities, we conducted linear regression models controlling for age and education to obtain adjusted means for each racial/ethnic group and used the “margins” and “pwcompare” commands in STATA for post hoc pairwise comparisons of differences in means from these models. We used education as a marker of socioeconomic status because it was missing for fewer respondents (three missing for education versus eight missing for income). We did not control for country of birth because it was strongly collinear with identifying as Latina or Vietnamese. For products or services that were used less frequently (<0.5 times per week), such as feminine hygiene products, certain hair products, and nail products, we compared the percent of women who used these products or services at least once per month using logistic regression to obtain age- and education-adjusted percents and *p* values for pairwise comparisons. Personal care products or services that did not differ significantly by racial/ethnic group in the linear or logistic regression models are not presented in tables but are noted in table footnotes. For the additional questions on reasons for purchasing, product use from other countries, and ingredients avoided, we calculated the number and percent of respondents and tallied open-ended responses of specific ingredients and products. An alpha level of 0.05 was set for statistical significance.

## Results

Our sample consisted of 70 Black women, 73 Latina women, 78 Vietnamese women, 79 White women and 18 Mixed Race women. The study population differed on several demographic characteristics by race/ethnicity (Table 1). Black women who participated were generally older and Latina women were younger. A majority of Black, White, and Mixed Race women were born in the United States whereas most Vietnamese women (93.6%) were born in Vietnam and almost half of Latina women (41.1%) were born in Mexico. Vietnamese women were most likely to have less than a high school education while White and Mixed Race women had the highest levels of education. White women also had the highest household income and Latina and Vietnamese women had the lowest.

**Table 1:** Demographic characteristics by race/ethnicity (CAPABLE Study, 2019–2020).

	Black	Latina	Vietnamese	White	Mixed race	
Characteristic	*n* = 70	*n* = 73	*n* = 78	*n* = 79	*n* = 18	*p* value
Age, median (IQR)	56 (27–47)	34 (24–40)	40 (32–48)	35 (27–47)	35 (32–54)	<0.001
Work in beauty industry, *n* (%)						
No	61 (87.1)	71 (98.6)	65 (86.7)	73 (93.6)	15 (83.3)	0.04
Yes	9 (12.9)	1 (1.4)	10 (13.3)	5 (6.4)	3 (16.7)	
Country of birth, *n* (%)						
U.S.	66 (95.7)	41 (56.2)	3 (3.9)	78 (98.7)	17 (94.4)	<0.001
Mexico	0 (0.0)	30 (41.1)	0 (0.0)	0 (0.0)	0 (0.0)	
Vietnam	0 (0.0)	0 (0.0)	73 (93.6)	0 (0.0)	0 (0.0)	
Other	3 (4.4)	2 (2.7)	2 (2.6)	1 (1.3)	1 (5.6)	
Education, *n* (%)						
Less than high school	3 (4.4)	8 (11.0)	24 (31.2)	0 (0.0)	0 (0.0)	<0.001
High school graduate/GED	23 (33.3)	26 (35.6)	25 (32.5)	20 (25.6)	5 (27.8)	
College degree	43 (62.3)	39 (53.4)	28 (36.4)	58 (74.4)	13 (72.2)	
Household income, *n* (%)						
$20,000 or less	6 (9.0)	16 (23.2)	11 (14.1)	8 (10.3)	5 (27.8)	<0.001
$20,001–$40,000	16 (23.9)	14 (20.3)	24 (30.8)	14 (18.0)	3 (16.7)	
$40,001–$60,000	16 (23.9)	13 (21.0)	21 (26.9)	9 (11.5)	3 (16.7)	
$60,001–$80.000	16 (23.9)	10 (14.5)	10 (12.8)	14 (18.0)	1 (5.6)	
$80,001–$100,000	8 (12.0)	8 (11.6)	5 (6.4)	4 (5.1)	2 (11.1)	
More than $100,000	5 (7.5)	8 (11.6)	7 (9.0)	29 (37.2)	4 (22.2)	

*IQR* Interquartile range.

### Makeup products

The most frequently used makeup items overall were lip balm (overall mean = 4.7 days per week), mascara (overall mean = 3.1 days per week), and lipstick (overall mean = 3.0 days per week). Distinct trends were seen by race/ethnicity (Table 2), with Latina women tending to use more makeup. Latina women wore mascara, eyeliner, eyebrow pencil, foundation, blush and all lip products (lip gloss, lipstick, lip stain, lip liner, and lip plumper) except lip balm significantly more often than women of other races/ethnicities after controlling for age and education. For many makeup items, Black and Mixed Race women used these products the least often.

**Table 2:** Age- and education-adjusted mean use per week of personal care products by race/ethnicity, with the highest mean per category in bold (CAPABLE Study, 2019–2020).

	Average days used per week (mean ± se)				
	Black	Latina	Vietnamese	White	Mixed race
	*n* = 70	*n* = 73	*n* = 78	*n* = 79	*n* = 18
*Makeup*					
Mascara	1.7 ± 0.3	**4.7** **±** **0.3**^**B, V, W, M**^	2.4 ± 0.3	3.7 ± 0.3^B, V, M^	2.2 ± 0.6
Eyeliner	1.8 ± 0.3	**3.0** **±** **0.3**^**B, M**^	2.6 ± 0.3^M^	2.2 ± 0.3	1.1 ± 0.6
Eyeshadow	0.4 ± 0.3	2.1 ± 0.3^B^	**2.2** **±** **0.3**^**B**^	**2.2** **±** **0.2**^**B**^	1.2 ± 0.5
Glue-on Eyelashes	0.4 ± 0.2^V^	0.6 ± 0.2^W, V^	**1.6** **±** **0.2**^**W, B, L**^	−0.1 ± 0.2	0.2 ± 0.4
Eyebrow tint/pencil	1.9 ± 0.4	**3.5** **±** **0.3**^**B, V, W**^	2.0 ± 0.3	2.4 ± 0.3	2.2 ± 0.7
Foundation (with SPF^1^)	0.8 ± 0.3	**3.9** **±** **0.3**^**B, V, W, M**^	2.8 ± 0.3^B^	2.4 ± 0.3^B^	1.6 ± 0.6
Concealer	0.7 ± 0.3	2.5 ± 0.3^B, M^	**3.4** **±** **0.3**^**B, L, W, M**^	2.7 ± 0.3^B, M^	1.0 ± 0.6
Highlighter	0.5 ± 0.3	2.3 ± 0.3^B, W^	**2.9** **±** **0.3**^**B, W, M**^	1.5 ± 0.3^B^	1.2 ± 0.6
Blush	0.6 ± 0.3	**3.7** **±** **0.3**^**B, W, M**^	3.5 ± 0.3^B, W, M^	2.4 ± 0.3^B^	1.4 ± 0.6
Lip gloss	**3.0** **±** **0.4**^**V, W**^	**3.0** **±** **0.3**^**V, W**^	2.0 ± 0.3	1.9 ± 0.3	3.0 ± 0.6
Lipstick	2.8 ± 0.3	**4.1** **±** **0.3**^**B, V, W, M**^	2.8 ± 0.3	2.5 ± 0.3	1.5 ± 0.6
Lip stain	0.3 ± 0.2	**1.2** **±** **0.2**^**B, V, W**^	0.3 ± 0.2	0.7 ± 0.2	0.8 ± 0.4
Lip liner	0.8 ± 0.3	**2.0** **±** **0.3**^**B, V, W, M**^	1.2 ± 0.3	0.7 ± 0.2	0.5 ± 0.5
Lip plumper	0.1 ± 0.1	**0.6** **±** **0.1**^**B, V, W**^	0.2 ± 0.1	0.0 ± 0.1	0.4 ± 0.2
Lip balm/chapstick	4.7 ± 0.3	4.0 ± 0.3	**5.0** **±** **0.3**^**L**^	**5.0** **±** **0.3**^**L**^	**5.0** **±** **0.6**
*Hair products*					
Shampoo	0.6 ± 0.2	**6.1** **±** **0.2**^**B, V, W, M**^	4.6 ± 0.2^B, M^	4.1 ± 0.2^B^	3.3 ± 0.4^B^
Conditioner	0.8 ± 0.3	**5.4** **±** **0.3**^**B, V, W, M**^	4.5 ± 0.2^B, M^	4.0 ± 0.2^B^	3.0 ± 0.5^B^
Leave-in conditioner	1.6 ± 0.2^V, M^	1.0 ± 0.2^V^	0.2 ± 0.2	0.9 ± 0.2^V^	**1.8** **±** **0.4**^**V, W**^
Gel/mousse	1.7 ± 0.3^V, W^	1.6 ± 0.2^V, W^	0.5 ± 0.2	0.8 ± 0.2	**2.5** **±** **0.5**^**V, W**^
Hair spray—pump	0.3 ± 0.2	**1.2** **±** **0.2**^**B, V, W, M**^	0.1 ± 0.2	0.4 ± 0.2	0.1 ± 0.3
Hair spray—aerosol can	0.1 ± 0.2	1.1 ± 0.2^B, V^	0.0 ± 0.2	**1.3** **±** **0.2**^**B, V, M**^	0.4 ± 0.4
Hair oil/grease	**2.6** **±** **0.2**^**L, V, W**^	1.6 ± 0.2^V, W^	0.0 ± 0.2	0.7 ± 0.2	2.2 ± 0.5^V, W^
*Skincare products*					
Makeup remover (liquid)	0.3 ± 0.3	**2.5** **±** **0.3**^**B**^	1.7 ± 0.3^B^	1.9 ± 0.3^B^	1.5 ± 0.6
Makeup remover (wipes)	0.8 ± 0.3	**2.7** **±** **0.3**^**B**^	2.1 ± 0.3^B^	2.0 ± 0.3^B^	2.0 ± 0.6
Toner	**2.2** **±** **0.4**^**L**^	0.9 ± 0.3	1.4 ± 0.3	1.7 ± 0.3	1.0 ± 0.6
Foaming facial cleanser/face soap	3.5 ± 0.4	4.3 ± 0.3	**7.2** **±** **0.3**^**B, L, W, M**^	4.1 ± 0.3	3.4 ± 0.6
Exfoliating cleanser	1.2 ± 0.3	2.0 ± 0.3	**5.2** **±** **0.3**^**B, L, W, M**^	1.5 ± 0.3	1.4 ± 0.6
Face scrub	0.8 ± 0.3	1.5 ± 0.3^W, M^	**3.3** **±** **0.3**^**B, L, W, M**^	0.8 ± 0.3	0.2 ± 0.5
Skin lightening cream	0.1 ± 0.1	0.3 ± 0.1	**0.5** **±** **0.1**^**W**^	0.0 ± 0.1	0.0 ± 0.3
Face cream (without SPF)	3.1 ± 0.4	2.4 ± 0.3	3.5 ± 0.3^L^	**4.1** **±** **0.4**^**L, M**^	1.9 ± 0.7
Face sunscreen	2.1 ± 0.4	**4.0** **±** **0.4**^**B**^	3.1 ± 0.4	3.1 ± 0.4	3.1 ± 0.7
Sunscreen for body	0.6 ± 0.3	**2.1** **±** **0.3**^**B, V, W**^	0.7 ± 0.3	1.3 ± 0.3	1.2 ± 0.5
Moisturizer or body lotion	**6.0** **±** **0.3**^**V, W**^	**6.0** **±** **0.3**^**V, M**^	4.5 ± 0.3	4.8 ± 0.3	5.7 ± 0.5^V^
Body, shea, or cocoa butter	**4.1** **±** **0.3**^**L, V, W**^	2.1 ± 0.3^V, W^	0.2 ± 0.3	1.0 ± 0.3^V^	2.1 ± 0.6^V^
Liquid soap or body wash	5.5 ± 0.3	5.4 ± 0.3	**6.6** **±** **0.3**^**B, L, W**^	5.3 ± 0.3	5.8 ± 0.5
Bar soap for your body	3.3 ± 0.4^V, M^	3.7 ± 0.3^V, W, M^	0.3 ± 0.3	2.5 ± 0.3^V^	1.5 ± 0.6
Shaving cream	0.5 ± 0.2	1.0 ± 0.2^V^	0.3 ± 0.1	**1.2** **±** **0.2**^**B, V**^	0.0 ± 0.3
*Deodorants and fragrance*					
Solid, stick, roll-on deodorant	**5.9** **±** **0.4**^**V**^	5.8 ± 0.3^V^	4.8 ± 0.3	5.6 ± 0.3	5.2 ± 0.6
Spray deodorant	**1.3** **±** **0.3**^**V**^	0.9 ± 0.2^V^	0.1 ± 0.2	0.9 ± 0.2^V^	0.6 ± 0.5
Perfume, cologne, or spray	**4.2** **±** **0.4**^**W**^	4.1 ± 0.3^W^	4.0 ± 0.3^W^	2.7 ± 0.3	3.1 ± 0.7
Body powder/baby powder	**0.7** **±** **0.2**^**V**^	**0.7** **±** **0.2**^**V**^	0.1 ± 0.2	0.4 ± 0.2	**0.7** **±** **0.4**
Essential oils	**2.3** **±** **0.2**^**L, V, W**^	0.7 ± 0.2	0.3 ± 0.2	0.9 ± 0.2	1.4 ± 0.5^V^

The following items did not differ by racial/ethnic group and are not included in the table: eyebrow tint, face masks, dry shampoo.

*SPF* Sun protection factor.

^B, L, V, W, M^Superscripts indicate that mean is significantly higher (*p* < 0.05) in pairwise comparison than mean for Black (B), Latina (L), Vietnamese (V), White (W), or Mixed-Race (M) women, respectively.

### Hair products

**Fig. 1:**
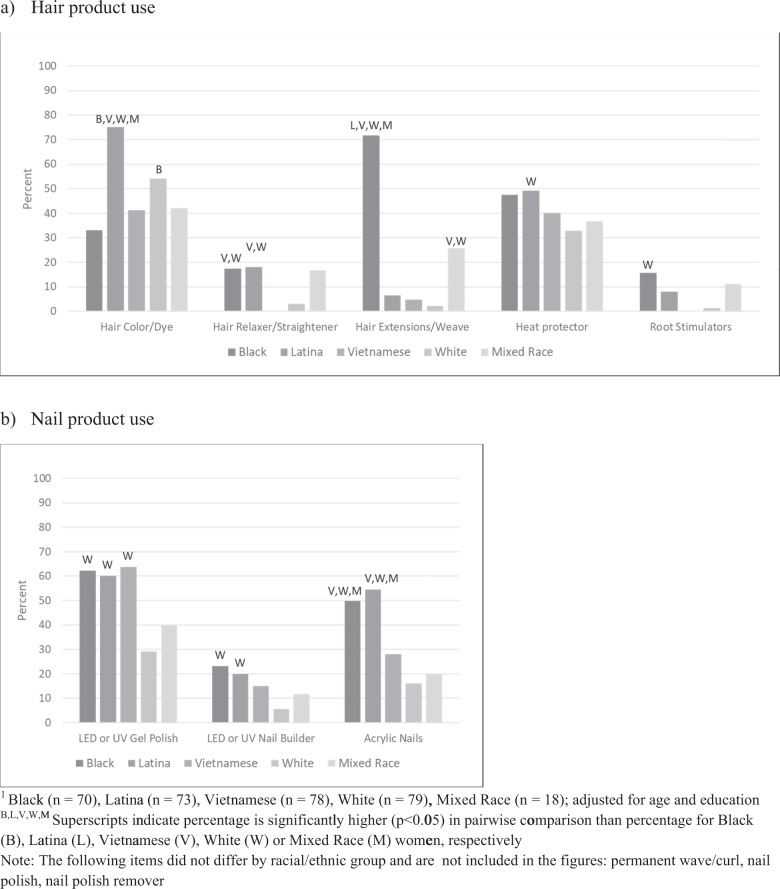
Percent of women reporting use of hair and nail products at least once per year, by race/ethnicity^1^. **a** Hair product use. **b** Nail product use ^1^Black (*n* = 70), Latina (*n* = 73), Vietnamese (*n* = 78), White (*n* = 79), Mixed Race (*n* = 18); adjusted for age and education. ^B,L,V,W,M^Superscripts indicate percentage is significantly higher (*p* < 0.05) in pairwise comparison than percentage for Black (B), Latina (L), Vietnamese (V), White (W), or Mixed Race (M) women, respectively Note: The following items did not differ by racial/ethnic group and are not included in the figures: permanent wave/curl, nail polish, nail polish remover.

Shampoo and conditioner were the most frequently used hair products (overall mean = 3.9 and 3.7 days per week, respectively), but there were differences by race/ethnicity (Table 2). Black women used shampoo and conditioner significantly less frequently than all other race/ethnicities. Mixed Race women were the most frequent users of leave-in conditioner and hair gel/mousse while White and Latina women were the most frequent users of hair spray (aerosol can or pump, respectively). Black women were most likely to use hair oil and root stimulators (Fig. 1a).

Although hair coloring was fairly common across all groups, Fig. 1a shows that Latina (75.1%) and White (54.2%) women most commonly used hair color at least once per year. The majority (71.6%) of Black women used hair extensions or weaves at least once per year, while less than 10% of White, Vietnamese, and Latina women did the same (Fig. 1a).

### Skincare products

The most frequently used skincare products overall were liquid soap/body wash (overall mean = 5.7 days per week), moisturizer/body lotion (overall mean = 5.3 days per week), and soap or foaming facial cleanser (overall mean = 4.7 days per week). Latina women were most likely to use makeup remover (Table 2), which is consistent with their more frequent use of many makeup products. Vietnamese women used facial products such as soap/foaming facial cleanser, exfoliating cleanser, face scrub, and skin lightening cream the most. Black women used body lotion and body/shea/cocoa butter most frequently.

### Deodorant and fragrance

Solid, stick, and roll-on deodorant products were commonly used among all races/ethnicities (mean = 5.5 days per week) as well as perfume, cologne or body spray (mean = 3.7 days per week). Black women used the most fragrance products overall, while Vietnamese women used most of these products significantly less often than any other racial/ethnic group (Table 2).

### Nail products

Nail polish and nail polish remover were used similarly by all races/ethnicities (not shown). However, Black and Latina women were much more likely to have acrylic nails at least once per year and Black, Latina, and Vietnamese women were most likely to use UV-cured gel polish or nail builder (Fig. 1b). White women were the least likely to use these nail products.

### Feminine hygiene products

The most commonly used feminine hygiene products were feminine wash/cleanser and feminine wipes, which were used regularly (i.e., at least once per month) by 29.6% and 30.1% of women, respectively. Table 3 shows that Mixed Race women were most likely to report regularly using douches (13.3%) and feminine wash/cleanser (40.0%), while Latina women were most likely to use feminine wipes (54.0%) and feminine sprays (16.9%). Black women were also quite likely to use these feminine hygiene products while White women were least likely to use them. Like White women, Vietnamese women rarely used most feminine hygiene products, with the exception of feminine wash/cleanser which was used by 30.5% at least once per month.

**Table 3:** Age- and education-adjusted percent using feminine hygiene products by race/ethnicity, with the highest percent in each category in bold (CAPABLE Study, 2019–2020).

	Used at least once per month				
	Black	Latina	Vietnamese	White	Mixed Race
	*n* = 70	*n* = 73	*n* = 78	*n* = 79	*n* = 18
Feminine wipes	29.8%	**54.0%** ^**B, V, W, M**^	19.8%	18.2%	25.6%
Feminine wash/cleanser	38.2%^W^	39.7%^W^	30.5%^W^	9 .4%	**42.0%**^**W**^
Feminine spray	13.8%^W^	**16.9%**^**W**^	8.7%	2.6%	11.4%
Vaginal douche	8.9%^V,W^	6.1% ^V, W^	0.0%	0.0%	**13.3%** ^**V, W**^

The following items did not differ by racial/ethnic group and are not included in the table: feminine powder/baby powder, lubricant.

^B,L,V,W,M^Superscripts indicate that mean is significantly higher (*p* < 0.05) in pairwise comparison than mean for Black (B), Latina (L), Vietnamese (V), White (W), or Mixed-Race (M) women, respectively.

### Products from other countries

There were significant differences by race/ethnicity in use of products brought from other countries (Table 4). Black women were most likely to have used products from other countries while White women were the least likely. Vietnamese women were the most likely to use products bought in the U.S. but that had a label in a language other than English (26.0%), followed by Latinas (14.1%). The most common regions of origin for imported products used by Black women were West Africa, particularly Ghana or Nigeria, and the Caribbean, and the most common imported products were shea butter and black soap. Vietnamese women were most likely to get products from Vietnam, Korea, and Japan, and to use products including face masks and skin lightener that did not have a label in English. Latina women were most likely to use products from Mexico, although they also reported using products from Korea and China.

**Table 4:** Use of products brought to the United States from other countries.

Ever use^a^	Black	Latina	Vietnamese	White	Mixed race
	*n* = 63–68^b^	*n* = 64–73	*n* = 77–78	*n* = 71–78	*n* = 18
Products brought from other countries, *n* (%)	14 (20.6)^L, W^	6 (8.2)	10 (12.8)	4 (5.1)	2 (11.1)
Products bought in U.S. with label not in English, *n* (%)	3 (4.8)	9 (14.1)	20 (26.0)^B, W^	6 (8.5)	0 (0.0)
Most common other countries	Africa/Caribbean Ghana Nigeria Brazil	Mexico China Korea	Vietnam Japan Korea	Russia Nigeria Israel France Germany Japan Korea	Africa/ Caribbean France Morocco

^B, L, V, W, M^Superscripts indicate that percentage is significantly higher (*p* < 0.05) in pairwise comparison than the percentage for Black (B), Latina (L), Vietnamese (V), White (W), or Mixed-Race (M) women, respectively.

^a^Questions asked: “Do you ever use products brought from other countries?” and “Do you ever use products bought in the U.S. with a label not in English?”

^b^Number of respondents differ by question due to participant non-response. For products from other countries, *n* = 69 Black, *n* = 73 Latina, *n* = 78 Vietnamese, *n* = 78 White and *n* = 18 Mixed-Race women. For products not in English, *n* = 63 Black, *n* = 64 Latina, *n* = 77 Vietnamese, *n* = 71 White, and *n* = 18 Mixed-Race women.

### Ingredients of concern

Concern about specific ingredients in products varied by race/ethnicity (Table 5). Black, Mixed Race, and White women were much more likely to state that they avoided certain ingredients than Latina and Vietnamese women. Only 13.9% of Latina women and 15.4% of Vietnamese women reported that they try to avoid certain ingredients in their personal care products, while over half of Mixed Race women and Black women surveyed did the same. Parabens and sulfates were mentioned by Black, Latina, Vietnamese, and White women as ingredients they avoid. Black, Latina, and White women also reported avoiding fragrance or perfume. Additionally, only 11.1% of Vietnamese women said they would use a fragrance-free product alternative if available, compared to 83.3% of Black women, 74.0% of Latina women, 72.9% of White women, and 83.3% of Mixed-Race women.

**Table 5:** Concern about specific ingredients in personal care products.

	Black	Latina	Vietnamese	White	Mixed race
	*n* = 60–62^a^	*n* = 50–72	*n* = 45–78	*n* = 59–78	*n* = 12–15
Avoid certain ingredients, *n* (%)	31 (50.0)^L, V^	10 (13.9)	12 (15.4)	33 (42.3)^L, V^	8 (53.3)^L, V^
Ingredients avoided^b^	Parabens Alcohol Fragrance/ Perfume Sulfates SLS Formaldehyde Aluminum	Parabens Sulfates Lead Fragrance/ Perfume	Parabens Phthalates Toluene Formaldehyde Sulfates Aluminum	Parabens Alcohol Sulfates Aluminum Phthalates Fragrance/ Perfume Dyes	None Mentioned
Would use fragrance-free alternative if available, *n* (%)	50 (83.3)^V^	37 (74.0)^V^	5 (11.1)	43 (72.9)^V^	10 (83.3)^V^

*SLS* Sodium laureth sulfate.

^B, L, V, W, M^Superscripts indicate that percentage is significantly higher (*p* < 0.05) in pairwise comparison than the percentage for Black (B), Latina (L), Vietnamese (V), White (W), or Mixed-Race (M) women, respectively.

^a^Number of respondents differ by question due to participant non-response. For avoiding certain ingredients, *n* = 62 Black, *n* = 72 Latina, *n* = 78 Vietnamese, *n* = 78 White and *n* = 15 Mixed-Race women. For using fragrance-free alternatives, *n* = 60 Black, *n* = 50 Latina, *n* = 45 Vietnamese, *n* = 59 White, and *n* = 12 Mixed-Race women.

^b^Ingredients reported by at least two participants.

Finally, most women from all races/ethnicities reported choosing products based on “what works well;” however, there were differences among the other factors taken into consideration for purchases. Most Black women said they chose products that were “made for my race” or labeled as “natural,” while most Vietnamese women said they chose products that were “the right price.”

## Discussion

We found distinct trends of personal care product use by race/ethnicity: Latina women used several makeup products more frequently compared to women of other races/ethnicities; Black women typically used certain hair products or styles, including extensions and hair oil more frequently but used shampoo and conditioner less frequently; Vietnamese women were most likely to use various facial cleansing products; and Latina, Mixed Race, and Black women most frequently used several types of feminine hygiene products. Black women were most likely to use products brought from other countries and Vietnamese women were most likely to use products that didn’t have labels in English. Additionally, Latina and Vietnamese women were both much less likely than Black, Mixed Race, and White women to try to avoid certain ingredients in their personal care products such as parabens, phthalates, sulfates, or fragrance.

Our findings support some of the patterns found by Wu et al. in a study of frequency of personal care product use among households in California [26]. They found that Black women were most likely to have their hair permanently treated (chemically straightened or relaxed), although were least likely to use shampoo and conditioner, which is consistent with our results. Additionally, they found that Asian women most commonly used skincare products; however, our study found that Vietnamese women most commonly used skincare products for the face, but not necessarily for the body. Wu et al.’s study population was majority White with <3% identifying as African American [26, 41]. Our study builds upon the trends observed in Wu et al. [26] while providing a more detailed characterization of products used by Black, Latina, Vietnamese, Mixed Race, and White women in particular communities of California.

Our results are also similar to many of the patterns found by Dodson et al. in the Taking Stock Study in California [39]. Both of our studies found that Black women often use hair products such as shampoo/conditioner less than women of other race/ethnicities and Latina women used some makeup products more frequently. However, we also saw some differences in patterns of use by race/ethnicity between the two studies. We reported overall higher usage of some feminine hygiene products and that Latina women were more likely to use some of these products, such as feminine wipes, whereas Dodson et al. [39] found that Black women used these products most frequently. Some of these distinctions may be due to the differences in design of the two studies. Our study surveyed specific communities in California while Dodson et al. conducted an online survey that was open to women throughout the state of California.

A previous study of feminine hygiene product use from NHANES data found that African American women used more vaginal douches, feminine spray, and feminine powder compared to non-Hispanic White and Mexican American women [34]. Our study showed that Latina, Black, and Mixed Race women commonly used feminine wipes, spray, and wash and that a large proportion of Vietnamese women are also using feminine washes.

Racial/ethnic differences in usage patterns of personal care products may be the result of a long history of racial discrimination and targeted marketing to women of color based on upholding White beauty standards (e.g., straight hair, lighter skin) [12]. These product use differences may explain different exposure patterns to several EDCs found in these products. For example, in the nationally-representative NHANES sample, African American and Hispanic women had the highest levels of methyl paraben and propyl paraben, preservatives that are commonly found in makeup and other personal care products, while non-Hispanic White women had the lowest levels [30]. Black women are the largest purchasers of many personal care products, and Latina women are the fastest growing group in this market [12]. The Latina women in our study were the most frequent users of makeup, which may result in higher paraben levels than other races/ethnicities in California. Douching has been associated with higher body burdens of diethyl phthalate [34] and dichlorobenzene [42], chemicals found in scented personal care products, potentially placing Black and Latina women at higher risk of exposure. Although only a small percentage of women in our study used skin lightening creams, they tended to be either Vietnamese or Latina. Because mercury has been repeatedly found in skin lighteners, Vietnamese and Latina women may be at a higher risk of exposure to mercury [37].

Our study also found that Vietnamese and Latina women were the least likely to intentionally avoid certain ingredients in their personal care products, such as parabens or phthalates. Additionally, most Vietnamese women, in contrast to all other racial/ethnic groups surveyed, reported that they would not choose a fragrance-free version of a product if it was available. Over 70% of all other races/ethnicities reported the desire to choose fragrance-free products if available. Because fragrance ingredients may be associated with endocrine disruption, cancer, and other health risks [43], avoiding fragranced products could be an important method to reduce risk. Through future label review and laboratory testing of specific products marketed in each population, we plan to further characterize exposure to potentially hazardous chemicals to better understand the specific exposures and disparities in different communities in California.

This study has several limitations. First, we surveyed a convenience sample of women from three specific communities within California and therefore we may not have a representative sample of women in California. There were a limited number of Mixed Race women in our sample, limiting our ability to make inferences about this group; however, we felt it was important that Mixed Race women be included as they are a growing population in the United States and are often excluded from other exposure studies. Mixed Race women can also identify in a myriad of different ways, making this category inherently heterogeneous, and therefore conclusions about this group may be less generalizable. Additionally, race is a social construct, and thus personal care product use patterns must also be understood in the broader social and cultural context. We also excluded those under 18 years of age in our study, and the median ages for each group were at least mid-thirties. There may be different findings among younger women and teens. Lastly, each community organization recruited women from different locations (e.g., churches, medical offices, etc.) which could influence the results. The strengths of the study are as follows. There have been very few studies to date that examine disparities in exposure from personal care product use, particularly in Latina and Asian women. Our study provides information on specific racial/ethnic groups in California by working with communities that may be likely to be otherwise overlooked. We used a community-informed questionnaire that obtained detailed information about use frequency and types of products used, which helps inform potential risk from exposure to chemicals in personal care products. In addition, our survey asked unique questions about product selection, including some open-ended questions such as which ingredients different groups try to avoid.

Information about frequency of product use is needed in order to estimate exposure and thus this research can be used for further risk assessment in order to better characterize the disparities in chemical exposure that Black, Vietnamese, Latina, and Mixed Race women may have compared to White women. Our data can also inform efforts to reduce exposure and risk, particularly in communities of color. Future research should continue to examine health inequities that may be due to differential chemical exposures and how they fit into the larger environmental and social context.
